# “Nine Dimensions”: A multidisciplinary approach for community engagement in a complex postwar border region as part of the targeted malaria elimination in Karen/Kayin State, Myanmar

**DOI:** 10.12688/wellcomeopenres.14698.2

**Published:** 2019-02-21

**Authors:** Decha Tangseefa, Krishna Monthathip, Naruemol Tuenpakdee, Andrea König, Ladda Kajeechiwa, May Myo Thwin, Suphak Nosten, Saw Win Tun, Kayin Ma, Ahmar Hashmi, Khin Maung Lwin, Phaik Yeong Cheah, Lorenz von Seidlein, Francois Nosten

**Affiliations:** 1 Center for Southeast Asian Studies, Kyoto University, Kyoto, 606-8501, Japan; 2 Faculty of Political Science, Thammasat University, Bangkok, 10200, Thailand; 3 Department of International Politics, Aberystwyth University, Aberystwyth, West Wales, SY23 3FL, UK; 4 Independent Researcher, Bangkok, 10160, Thailand; 5 Peace Information Center, Faculty of Political Science, Thammasat University, Bangkok, 10200, Thailand; 6 Shoklo Malaria Research Unit, Mahidol-Oxford Research Unit, Faculty of Tropical Medicine, Mahidol University, Mae Sot, 63110, Thailand; 7 Mahidol-Oxford Research Unit, Faculty of Tropical Medicine, Mahidol University, Bangkok, 10400, Thailand

**Keywords:** Community Engagement, Targeted Malaria Elimination, Myanmar-Thailand Border, Postwar, Cross-border

## Abstract

**Background:** In light of growing antimalarial drug resistance in Southeast Asia, control programmes have become increasingly focused on malaria elimination, composed of mass drug administration coupled with prompt diagnosis and treatment of symptomatic cases. The key to a successful elimination programme centres on high participation rates in targeted communities, often enhanced by community engagement (CE) efforts. Social science research was conducted to develop a conceptual framework used for CE activities in the Targeted Malaria Elimination programme, as a cross-border operation in Karen/Kayin State, Myanmar.

**Methods:** Data was collected from three main sources: (1) participant observation and semi-structured interviews of CE team members; (2) participant observation and semi-structured interviews with villagers; and (3) records of CE workshops with CE workers conducted as part of the TME programme.

**Results:** Interviews were conducted with 17 CE team members, with 10 participant observations and interviews conducted with villagers and a total of 3 workshops conducted over the course of this pilot programme in 4 villages (November 2013 to October 2014). Thematic analysis was used to construct the nine dimensions for CE in this complex, post-war region: i) history of the people; ii) space; iii) work; iv) knowledge about the world; v) intriguing obstacle (rumour); vi) relationship with the health care system; vii) migration; viii) logic of capitalism influencing openness; and ix) power relations.

**Conclusions:** Conducting CE for the Targeted Malaria Elimination programme was immensely complicated in Karen/Kayin State because of three key realities: heterogeneous terrains, a post-war atmosphere and cross-border operations. These three key realities constituted the nine dimensions, which proved integral to health worker success in conducting CE. Summary of this approach can aid in infectious disease control programmes, such as those using mass drug administration, to engender high rates of community participation.

## Introduction

Artemisinin-based combination therapies are globally the first line of treatment against *Plasmodium falciparum* malaria. The parasites, however, have become more resistant to artemisinin, posing a threat to malaria control. Resistance to antimalarial drugs has developed in Southeast Asia, with artemisinin resistance first reported in western Cambodia ( Dondorp *et al*., 2009) and spreading to other areas of mainland Southeast Asia ( Ashley *et al*., 2014). This is of great concern because resistance—as has happened with other antimalarial drugs—could spread to Africa at great cost to public health and malaria control efforts ( von Seidlein & Dondorp, 2015).

Myanmar has the highest prevalence of malaria in the Greater Mekong Subregion (GMS), including in the Karen/Kayin State along the Myanmar-Thailand border ( World Health Organization, 2015b) ^1^. Even though malaria transmission is low in the Karen/Kayin State, Myanmar, there is a significant reservoir of submicroscopic parasitemia in otherwise healthy people. The area is, therefore, at the vanguard in the battle against emerging drug resistance ( Phyo *et al*., 2012).

In the absence of more promising plans to contain *P. falciparum* malaria, a multipronged approach that includes mass drug administration (MDA) has been attempted ( von Seidlein & Dondorp, 2015; World Health Organization, 2015a). This requires simultaneously dispensing treatment to an entire population in a given geographic area ( Dial *et al*., 2014)—in communities where the proportion of asymptomatic, infected individuals is high—to complement conventional control methods such as early detection and treatment of clinical cases and vector control measures. Success needs to elicit a high participation of villagers to rapidly eliminate the reservoir of parasites and protect against new infections for an extended period following MDA programmes ( Adhikari *et al*., 2016; Newby *et al*., 2015; Okell *et al*., 2011; Sahan *et al*., 2017; von Seidlein & Greenwood, 2003; White *et al*., 2009).

To ensure high coverage rates and adherence, researchers have employed various community engagement (CE) activities in MDA trials ( Pell *et al*., 2017). This is particularly true of the Targeted Malaria Elimination (TME) programme across the GMS in Cambodia, Lao PDR, Myanmar, the Myanmar-Thailand border and Vietnam. As an integral part of the MDA component of TME, CE has involved five key elements that have been tailored to each respective site: 1) stakeholder and authority engagement; 2) enlisting local human resources; 3) utilizing formative research prior to conducting MDA trials; 4) responsiveness and adapting to local challenges as they arise; and 5) sharing control and leadership with the community in deciding and organizing activities ( Adhikari *et al*., 2017). In a systematic review of CE activities for mass antimalarial administrations, these broad categories were addressed through activities such as health education and provision of incentives, use of existing community structures and health infrastructure, mobilizing human resources, and government collaboration ( Adhikari *et al*., 2016). In the TME programme, these activities took the form of workshops, meetings and household visits with community leaders and members; exhibitions and educational activities targeting communities (including children); provision of ancillary care along with study related clinical monitoring; awareness campaigns through local media; enlisting village and community health workers for study implementation; and local theatre and drama programmes ( Adhikari *et al*., 2017; Adhikari *et al*., 2018; Kajeechiwa *et al*., 2016; Kajeechiwa *et al*., 2017; Lim *et al*., 2017; Nguyen *et al*., 2017; Pell *et al*., 2017).

These CE activities have been incorporated into the TME programme to engender community participation amidst an ongoing discussion as to what constitutes 'community' or 'participation' in the existing literature from low resource settings ( Atkinson *et al*., 2011; King *et al*., 2014; Tindana *et al*., 2007). Although the CE activities often follow systematic implementation and rigorous frameworks ( Adhikari *et al*., 2017; Lavery *et al*., 2010), there is a lack of an overarching conceptual framework for CE. Along the Myanmar-Thailand border, the TME team performed formative research to better articulate concepts necessary for tailored CE activities in this region. This research determined nine basic notions important for conducting cross-border CE in heterogeneous and postwar terrains ^2^. These key basic notions are: history of the people, space, work, knowledge about the world, intriguing obstacle (rumour), relationship with the health care system, migration, logic of capitalism influencing openness, and power relations. Of note, the authors point out that the logic of capitalism has been separated out to allow the inclusion of migration as an additional dimension, in contrast to the eight dimensions cited previously ( König *et al*., 2018). The authors summarise the approach used to create this conceptual framework, detailing the methods used to acquire a deeper understanding of the material conditions, socio-cultural contexts and political entanglements that led to creating effective CE activities in the context of malaria elimination.

## Methods

### Contextual background

In the context of the Myanmar-Thailand border, the TME project traversed a state-boundary, heterogeneous terrains and a postwar atmosphere of over half a century of armed conflict *and* militarized government ^3^. Awareness of the particularities of people, place and of the power dynamics at play became imperative for CE team members. Such a postwar border comes replete with particularly sensitive and vulnerable situations that pose ethical challenges for any public health intervention ( Parker, 2012). Specific to the Myanmar-Thailand border, villagers’ participation is complicated by histories of ethnic strife, armed conflict and military government in Myanmar ( Tangseefa, 2003; Tangseefa, 2006; Tangseefa, 2016). The peoples living along the border also show significant ethnic, linguistic, religious and political differences ( Scott, 2009)—although to a lesser degree than that of the whole of Myanmar ^4^. Many are either internal migrants or returnees from a string of “refugee camps” on the Thai side ( Kusakabe & Pearson, 2013; Phyo *et al*., 2012; Tangseefa, 2006; Tangseefa, 2016) ^5^. Moreover, how far a target village is from a state-boundary greatly affects any cross-border CE activities: location along a border and on the other side of the boundary renders such CE operations significantly different from other locations deep inside a nation-state where an operating team legally belongs. Any CE team faced with these realities hopes to lessen complex obstacles resulting from remnants of war pervading along such borders. Moreover, the Myanmar-Thailand border is a postwar area where coverage, utilization and technical quality of facility-based health care have been grossly deficient ( Low *et al*., 2014). Constraints in accessing health services are intensified due to lack of infrastructure, limited access to resources and displacement of people ( Teela *et al*., 2009).

Given this context, the TME programme beginning in 2013 was an historic and greatly challenging undertaking. With just over two years after the first semi-democratic election on November 7, 2010—since the coup d'état in 1962—there was no certainty that Myanmar’s “openness” would be sustainable. Decades of armed conflict and militarization presented immense infrastructural and logistical obstacles in accessing many villages in Myanmar. Importantly, this reality also left strong traces of violence and authoritarianism manifested as: local communities harbouring enmity toward parties who had committed violence; a strong distrust towards outsiders; and a pre-democratic, bureaucratic personnel still largely intact. These realities played out against a backdrop of long-standing memories of mutual animosity among Burman (Bamar) and Karen peoples both inside the country and along the border ( Tangseefa, 2003; Tangseefa, 2006; Tangseefa, 2016). When taken together, all of these issues complicated achieving the aim of TME: a focused *elimination* of malaria given the *urgency* in preventing drug resistance spread ( Landier *et al*., 2017; Parker *et al*., 2017; Thu *et al*., 2017; von Seidlein & Dondorp, 2015; World Health Organization, 2015a). Moreover, MDA, as a testable tool for TME, requires wide-scale drug administration within a short timeframe ( Landier *et al*., 2017; Parker *et al*., 2017; Thu *et al*., 2017; von Seidlein & Dondorp, 2015; World Health Organization, 2015b). The sum of these factors forced a shorter, more focused engagement process contrary to recommendations for long-term engagement for infectious disease control ( Kaneko *et al*., 2014; McNaughton, 2012). Conditions for engagement were, therefore, far from optimal.

### Deriving the nine dimensions

Given this unique contextual background, the research team employed a qualitative approach to explore the TME CE team experiences to derive the nine dimensions. This research was conducted over the first 2 years of the 3-year pilot project along the Myanmar-Thailand border (2013–2016). The project began in 2013 by the Shoklo Malaria Research Unit (SMRU), an organisation that has been providing health services to border region populations since 1986. During the study period, the TME programme operated in four pilot villages, which exemplified the diversity of settlements in the Karen/Kayin State, Myanmar.

The villages—anonymised as A, B, C, and D—served as clear examples of this heterogeneous postwar border: varying with respect to proximity to urban areas, cultural homogeneity, villager mobility, and in the proportion of the population that are resettled migrant workers or “refugees”. Although located on a trading route, Village A is the farthest of all villages from an urban area, with many villagers having been uprooted by violence multiple times. Many of the displaced Karen villagers have since resettled and, under more recent control by the Democratic Karen Benevolent Army (DKBA), formerly of the Democratic Karen Buddhist Army), Sgaw and Pwo Karen families have also migrated into the area for agricultural work. Originally a farming village, Village B in the late 1990s became the site of saw mills as part of the growing lumbering industry along the border that became home to migrant workers up until 2002. Being near the border with good access to the Mae La Refugee Camp, Thailand, Village B has seen a recent influx of former refugees given more political openness in Myanmar and in light of rumours that camps on the Thai side may close soon. In addition to its history as a site for the lumbering industry, Village C has also been greatly affected by the armed conflict. Formerly under the Karen National Union/Karen National Liberation Army (KNU/KNLA) until the fall of Manerplaw (1995), Village C came under the control of the Democratic Karen Buddhist Army, which set about re-establishing many of the displaced villagers as agriculture resumed and saw mills reopened. In addition to attracting migrant workers and displaced settlers, refugees have also settled in Village C given its proximity to the refugee camps on the Thai side and is considered by the CE team to be highly mobile. Although Village D has the easiest access to urban areas, it shares a similar history with Village A in suffering repeatedly over the course of the extended armed conflict along the border. In contrast, however, the displaced populations have yet to resettle in Village D, and instead it is now made up of migrants (seasonal workers) as well as small business owners that represent Burman, Sgaw and Pwo Karen ethnicities.

The study field research team comprised three sets of people: the senior CE team, a social scientist (D.T.) and three social science research assistants. The CE team was part of the overall TME team, which also included: a logistics team managing supplies and transport; a survey team; and an information technology team responsible for GPS mapping and participant registration. These teams worked in concert to conduct key TME activities such as antimalarial drug distribution; blood sampling surveys; provision of basic health care; and monitoring migration/movement of participants. Apart from conducting their CE activities, the senior CE team members were also the team’s key field researchers. These dual duties were pivotal for two reasons: temporal imperative and cultural similarity. In terms of temporal imperative, aside from the strict time constraints of the programme, the team’s excursion into the Karen/Kayin State encountered tremendous logistical challenges because of its inception within 2 years of Myanmar’s first successful election since the coup d'état in 1962. Comprised of ethnic Karens and one ethnic Burman, the cultural similarity of the CE team to most peoples living in the four villages reduced the gap between the CE team (as outsiders) and the villagers.

While senior team members had many years of experience living and working along this border region, the CE team also included junior team members in their twenties with limited experience working in this region. Since the senior members had not been academically trained to conduct social science research, our team assigned three research assistants—each during a different period—to conduct participant observation and a series of in-depth interviews with all CE team members and villagers. Because of their cultural similarity to the target villages, senior team members acted as cultural mediators for the three Thai research assistants, deepening data quality and the subsequent development of the nine dimensions. Moreover, because the younger CE team members were not fluent in English, our interviews with them were facilitated and translated by the senior members. Once deemed appropriate for research assistants to travel to the four villages, each spent time interviewing villagers with senior team members as their interpreters ^6^.

Thus, the CE team’s experiences form the core of this study. Data comprised of interview and field notes and audio recordings were collected from three main sources: (1) semi-structured interviews of CE team members ( Supplementary File 1 contains the guide used for the interviews); (2) participant observation and semi-structured interviews with villagers; and (3) records of CE workshops with CE workers conducted as part of the TME programme. The interviews with CE workers included open-ended questions on their perceived roles; target village characteristics; their interactions with the villagers and activities they conducted; and observed barriers to participation. The research assistants joined the CE team during their work in the target villages between May and November 2013 and May and October 2014, spending 3 days in each village observing the CE activities as well as village life more generally. Interview and field notes were compiled and audio recordings were translated and transcribed. Inductive analysis of recordings was performed by D.T. D.T. summarised patterns in theory and conceptualization of CE work. This analysis was an iterative process—interviews, data collection, analysis—and was performed a total of three times over the course of this study. D.T.’s preliminary analysis directed subsequent exploration of relevant informants (CE team members and/or villagers) via interviews conducted by research assistants. Final thematic analysis of concepts was performed following completion of data collection, with the “9 Dimensions” initially drafted by D.T. and reviewed and approved by the CE and research teams for use as a framework to guide CE work. For the preparation of this manuscript, D.T. and A.H. facilitated a line-by-line review of the 9 Dimensions draft with the CE team to review the analysis, corroborate salient results and finalise a draft appropriate for publication.

### Ethical considerations

Oral informed consent was obtained from all CE workers for interviews and observations. Oral consent was chosen over written consent due to the potentially intimidating aspects of written consent for participants with personal histories of interrogation in a former war zones, both for villagers as well as CE staff alike. This consideration allowed for an environment more conducive to conversation, allowing better quality data. Interview transcripts and field note observations were anonymised, numbered, and saved on a password-protected computer. The study was approved by the Oxford Tropical Research Ethics Committee (OxTREC 1015-13, dated 29 Apr 2013) and received local approval through the Tak Community Advisory Board (T-CAB) and village committees in each of the four villages where the study took place.

## Results

### Nine dimensions

Interviews were conducted with a total of 17 CE team members (n=17), with 10 participant observations and interviews conducted with villagers (n=10). All interviews were conducted from May to November of 2013 and May to October of 2014. A total of 3 workshops (n=3) were conducted over the course of this project. Through thematic analysis, conceptual construct of nine dimensions for CE emerged.

This examination of CE strategies demonstrated the team’s awareness of the particularities of people, place and power dynamics at play in different locations. This gave weight to villagers’ quotidian lives situated “in-between” the two nation-states and social and political strife caused by decades of civil war ^7^. CE workers recognized three aspects of the target villagers’ lives: *worldview*, *time* and *desire*. In other words, how did the villagers view their worlds and the world at large? Did villagers have time to participate in the TME programme? Did villagers desire to participate? In answering these basic questions, the following nine dimensions emerged as a crucial conceptual tool for navigating CE: i) history of the people; ii) space; iii) work; iv) knowledge about the world; v) intriguing obstacle (rumour); vi) relationship with the health care system; vii) migration; viii) logic of capitalism influencing openness; and ix) power relations.

*i) History of the people*.

CE workers attempted to understand first, *who* villagers were during the time of engagement and second, *how* villagers had become who they were. Peoples’ ethnic, religious, educational, work and migratory backgrounds all constituted villagers’ worldviews and affected villagers’ senses of time and their willingness to participate in the TME programme. Knowing such aspects became crucial and CE workers identified ways in which histories influenced senses of belonging in target villages. Histories of places and peoples influence a community’s senses of belonging and obligations to those within the community, or to others, outside their communities (Cf. Harmanşah, 2015; Navaro-Yashin, 2012; Tangseefa, 2003; Tangseefa, 2006; Tangseefa, 2016). Two forms of history proved particularly important to the CE team: histories of violence and histories of cultural difference.

***Histories of violence*.** All four target villages were former war zones. Competing figures of sovereign power had rendered people’s lives in these rural areas even more vulnerable by the time the CE workers started TME. Some villagers—such as those in villages B and C—had resided in war zones until arriving in the village, where they took up a more permanent residence. Given this context, many villagers had been isolated and neglected by the outside world. The only outsiders many villagers had encountered during times of instability were affiliated with the armed conflict (e.g., soldiers). These experiences resulted in villagers developing a strong sense of distrust toward outsiders. As a CE team member reports from a resident of Village A:

“Years ago, the Burmese troops entered the village and took her husband as a porter. While working, he stepped on a landmine and lost his right foot. When some persons took her injured husband back to the village, she confronted the Burmese troops...[and complained]...in tears: 'How could you do this to us, we were a young couple, if anything happened to him, how could I live?' she said while also was out of breath. Moreover, she herself was still breastfeeding. Finally, because of her complaints and persistence, they took her husband to a nearby hospital and he survived. But now, when new outsiders come [sic] to the village to do work, she suspect [sic] of their intentions and their willingness to help her in times of emergency or need”. (CE Team member)

With such traumatic memories of prolonged violence and neglect, many villagers lacked a sense of belonging with outsiders and were not sympathetic to outside concerns, such as a medical endeavour and its promised benefit to people at large. Many Village A residents, therefore, did not participate in TME programme activities such as MDA and blood sampling surveys. Higher rates of participation were achieved in Villages B and C where the programme was seen as beneficial.

***Histories of cultural difference*.** CE workers learnt that it was essential to characterise how diverse each village is in terms of class, ethnicity, religion and political affiliation, among others. The more diverse a community, the more it was heterogeneous and, the less likely the villagers were to develop a deep sense of belonging. For example, Village D lacked a strong sense of belonging or a desire to benefit the community at large, while espousing broader worldviews compared to a homogenous community like Village A.

*ii) Space*

Space shapes peoples’ worldviews (Cf. Carter, 2002; Carter, 2013; Harmanşah, 2015; Lefebvre, 1991; Navaro-Yashin, 2012). CE workers heeded two aspects related to space: geographic accessibility of each target village and villagers’ ability to exchange with or access information from the outside world. Moreover, limited geographic access meant reduced mobility into and out of the village. Fewer visitors meant less access to pluralistic worldviews. Geographic isolation could also limit an outsider’s ability to communicate with the villagers through telecommunications requiring significant infrastructure. For instance, Village A was located deep in the jungle and far from the Myanmar-Thailand state-boundary. Villages B, C and D were located closer to roads and to the boundary. In general, Village A’s spatial isolation affected villagers' openness to outsiders and new ideas, including the TME programme. One TME team member stated:

“Village A is more difficult than other villages….because the villagers here are very…traditional. Once they believed something, they would never change their thoughts easily. So, it’s very difficult for our work….Even though we tell the truth to them, they wouldn’t believe or trust us easily because we are outsiders.” (TME team member)

Village A’s location, thus, shaped the villagers’ perspectives on medical practices in comparison to villagers from the other target villages. The CE team characterized Karens from Village A as “hill Karens” and the Karens from the other three as “lowland Karens”. A CE team member articulated:

“Hill Karens are not familiar with the idea that ill persons have to go to see a doctor. For them, normally when a person gets sick, mobile traditional healers could come and treat the patient at home. As for lowland Karens who live along the borders, [they]…would have more diverse experiences, because they are acquainted with living among peoples from different places. Therefore…[the lowland Karens] are more familiar with modern medication. If we explain the advantages and disadvantages of modern medicines or give them this knowledge, they would listen to us and understand”. (CE team member)

*iii. Work*

As work tended to occupy most villagers’ time, CE workers responded by scheduling their activities to suit villagers. One of the CE team members stated:

“During the TME activities in Villages A, B, C and D, we needed to adjust our time according to the available time of each village. Because villagers have different types of work and their available time. It was a challenge; [our] big team has their own routine, [their own] system...many of…[the team members] didn’t want to change but they need [sic] to change, [therefore] some quitted [sic]….Moreover, everyone [in the whole team] was still new in that context and some quitted [sic]….Hence, [it was important] for us to negotiate among ourselves that availa[bility] of people’s time is…important…[for the whole team to reach] our goal”. (CE team member)

When investigating work, CE workers evaluated two components: *duration* and *types* of work. Duration of work concerned working hours as they fluctuated either daily or seasonally. Many villagers relying on seasonal farming had to live in shelters on their farms outside the target villages. By noting which villagers were away due to seasonal farming, CE workers could plan MDA and community activities to accommodate the villagers’ schedule.

The main forms of work encountered included subsistence or commercial farming, unskilled labour, shopkeeping or seasonal gathering. This category also included unpaid work such as household chores and social responsibilities. Inquiring about types of work helped CE workers understand how particular kinds of work affected villagers’ availability and allowed adapting TME activities to fit villagers’ schedules. While inquiring about villager’s use of time, CE workers made sure to inquire across genders and types of occupations. The CE team also considered schedules potentially deviating from community norms—e.g., gamblers. Assessing marginal perspectives enabled the programme to reach as many people in the community as possible. Learning a variety of perspectives of how villagers come to terms with their worlds—such as for the “work” dimension—was central to CE work and understanding other dimensions.

*iv. Knowledge about the World*

CE workers set about understanding the breadth of worldviews present in each village. Worldviews were integral to how a community understood TME activities and how likely they were to participate.

In contrast to Village A, Villages B and C were more accessible. Many of the residents in Villages B and C had resettled from Mae La, a so-called “refugee camp” on the Thai side and were still registered in that shelter area. Individuals in the latter category traversed back and forth between the two countries and enjoyed registrations both as Myanmar citizens and shelter area residents so as to receive social welfare allotted to citizens while simultaneously receiving “camp” residents’ rations. Unlike most people growing up in Karen/Kayin State, villagers from Villages B and C had been exposed to some formal education, biomedical health care, varieties of organisations and the “camp’s” cosmopolitan atmosphere—lending a greater inclination to participate in the programme, as one of the TME senior team members reported:

“Village A is traditional…[the] village [is] located in [a] very remote and isolated areas [sic]. The village had been torn apart 3 times because of armed conflict. [Being] torn apart by war, its health and education [systems] badly destroyed, the villagers…[were]...left alone by [the] outside world. Given this, it is hard to talk about the modern medicine to some...villagers who may not have been told of these ideas in the past. The villagers were relying on traditional healer. For example, the villagers’ believed that pregnant woman [sic] could deliver a baby safely if the traditional healer prepared sacred water, candles from beeswax and conducted a special ceremony….Not only for Village A, other villages B, C, D also believed the same. The sacred water [is] used for injury, mumps, fishbone stuck in the throat etc…”. (TME senior team member)

*v. Intriguing obstacle (rumour)*

CE workers learnt to remain vigilant for intriguing obstacles for project implementation, with rumours being the most intriguing obstacle in this context. Because of many villagers’ limited worldviews regarding the changing world—in some villages more so than others—CE workers learnt that ineffective communication led to rumours and misperceptions about TME activities. To ensure greater understanding of TME to limit rumours and subsequent low participation, the CE workers disseminated culturally appropriate and medically accurate information about TME activities, such as MDA and blood survey sampling, in a form adjusted to language accessible to the villagers. To achieve this, CE workers identified information and communication methods most compatible with the target villagers’ material, cultural, and political conditions by integrating the villagers’ social, cultural, and political understandings in the disseminated information. One CE senior team member stated:

“There were many rumours circulating in Village D such as selling blood, providing mosquito nets in exchange for their blood and testing new medicines. These rumours became one of the major obstacles to the work of our staff and made the villagers doubt or distrust our TME activities. For instance, when we conducted the group discussions, we got information from some of the villagers suspected [sic] that our TME team drew their blood in order to sell for money. Also during blood sample surveys, villagers raised the questions…[whether or not] we [had] sold the blood to...BKK [Bangkok] and [had] got a lot of money”. (CE senior team member)

*vi. Relationship with the Health Care System*

CE workers investigated the villages’ relationships with health institutions to explore how these relationships influenced villagers’ utilisation of health care. The team also explored villagers’ senses of trust and loyalty toward respective health organisations. As one senior CE team member put it:

“Whenever we asked where they went when they were unwell, the villagers replied that they self-treated themselves or went to ‘quack[s]’ (fake doctors). If they had not gotten better they would have gone to some Thai health centres or nearby SMRU clinics or Mae Tao Clinic [all on the Thai side of the border]. All the four villages had different difficulties both [in terms of] geography and risks of crossing the border...usually patients arrived to [those] clinics [when they were already in]...a critical stage….[Moreover] a village ‘quack’ at Village A [once] told us: ‘people trust me because during fighting time there was no one provid[ing] medical supplies for villagers. I was lucky; a Karen lady [had] sen[t] me [a] small amount of medicine and with that I could treat villagers”. (CE senior team member)

Villagers isolated in times of need and those with perceived or real neglect from health institutions were more likely to be self-reliant and less likely to have an affiliation with any health care provider. Communities with more positive experiences with health institutions were likely to be more accustomed to modern biomedical practices and proved easier to convince to participate in MDA. However, loyalty towards a certain health care provider could also decrease the likelihood of villagers to accept health care from an unfamiliar health organisation.

Knowing villagers’ prior experiences with health organisations—including aspects of trust and/or loyalty towards the latter—proved vital to the success of the programme. If villagers had been isolated during hard times and felt neglected by health institutions, they were less likely to have any meaningful relationship with the latter. However, places where a health care provider had been present during challenging times instilled a sense of obligation within villagers toward these health institutions or a perceived need to participate in order to ensure future health service provision. This sense of belonging enabled the CE team to develop a trusting relationship with some villagers. However, villagers affiliated with other health care providers could be less interested in engaging with SMRU employees. In Village D, for instance, villagers already had access to different health care providers. If they were satisfied with those services, they would be less inclined to participate in SMRU’s activities. Finally, some villagers had had negative experiences with some health institutions in the past and carried with them the notion that engaging with a new provider could be risky.

*vii. Migration*

The CE workers were aware that the historic election on November 7, 2010 was the dawn of a migratory period within Myanmar. The election resulted in the country becoming more democratic, which resulted in more freedom of movement. Hence, more people had moved into Karen/Kayin State to improve their economic conditions. Among the four villages, Village D was the most obvious example of this shift towards internal migration. A variety of peoples had migrated seeking economic opportunities. This also had the effect of greater fragmentation observed in Village D with smaller, heterogeneous communities. Villagers often had financial means allowing them to meet health needs independent of the free services offered by SMRU or other international nongovernmental organisations. This heterogeneity and self-sufficiency resulted in a lack of a sense of belonging that affected the village's sense of “community” as a whole or perceived need for SMRU services or the TME programme. Village D’s migratory complexity led one CE team member to reflect:

“There are two basic types of migrants in Village D: IDPs [internally displaced persons] and economic migrants. These economic migrants require extra attention to enhance their participation. And there are two types of economic migrants: seasonal workers and businesspersons. During the MDA activity, it is difficult to get the seasonal workers to participate regularly for medication: for three days in each month and for three months in a row. Even if the seasonal migrants were willing to participate, it is very hard to do home-visit with them every two weeks because most of them don’t have houses. [As for] the businesspersons, they came from Myanmar’s inner area to run grocery shops and some of them also own lands. They focus more on their business and they don’t want to participate even though they understand about the CE messages. Because they can afford to pay for medicine and do not want to lose time to participate...because of their business interests. Also, the businesspersons and other villagers who own houses are unhappy if seasonal migrants are recorded by our ‘registration system’ in their house numbers. Because the house owners consider that the seasonal migrants do not belong to their villages”. (CE team member)

*viii. Logic of capitalism influencing openness*

CE workers learnt to pay more attention to the ways and the extent to which capitalism transforms everyone dictated by one of its logic—namely, to be open to all forms of activities as long as they make monetary profit (Cf. Appadurai, 1996; Appadurai, 2013; Bear, 2016; Jameson, 1992; Kocka, 2016; Tangseefa, 2003). CE workers, thus, examined how monetary profit affected the target community by exploring: who brought money into the community; where the money came from; and who were controlled by such financial capital. CE workers, thus, learnt that villagers influenced by such capital logic tended to be more tolerant and have broader worldviews—no matter where money came from and regardless of their class, ethnic, religious or political backgrounds ^8^. Through experiences working with diverse groups, these villagers tended to have a “more open” worldview that was receptive to new or different ideas. However, this group of people might not have necessarily wanted to participate in MDA because of limited time and their focus on monetary benefits. In the process, these villagers’ lives were advanced because their individual entrepreneurial freedoms and skills had been liberated, i.e., their lives had both been capitalised and neoliberalised (Cf. Harvey, 2007; Steger & Roy, 2010).

Among the four villages, Village D was the most neoliberalised and capitalisation of both space and time were the rule. One senior TME team member stated:

“Business persons who run grocery shops and gambling shops in the village are the most difficult group to gain their participation….It’s not that they don’t understand our project. They have basic modern medical knowledge. They can understand the seriousness [of malaria and drug-resistant malaria]. But they just have no time for our programme…If they become ill, they can take care of themselves because they have money to go to private clinics. So, these people don’t feel that they owe a debt of gratitude to SMRU and, therefore, don’t feel belong to SMRU”. (CE senior team member)

*ix. Power Relations*

For CE workers, it was important to understand power relations within, as well as surrounding, the target villages. Additionally, the workers learnt to map the terrains of competing figures of sovereign power—be they ethnic armed groups, Myanmar’s state apparatuses or international nongovernmental organisations—that could wreak havoc to the already fragile populations.

Both internal and external political entanglements interweaved with each village’s social fabric to influence villagers’ decision to participate in the TME. Moreover, like former war zones throughout the world, the Myanmar-Thailand border had a complex history of multiple organisations working with or competing against one another ( Parker, 2012). Organisations engaging with the TME programme, thus, represented a variety of working methods and espoused political viewpoints that could lead to tension and misunderstanding. Within this web of relationships, the CE workers learnt to be cognizant of two aspects of power relations to ensure programme success: first, *local leadership*; and second, *power relations at both micro and macro levels*.

Both informal and formal local leaders can act as “gatekeepers” to communities ( Fountain *et al*., 2007). Within each village, health workers met influential informal leaders, such as monks, teachers, pastors, some elders, traditional healers, local medics, as well as formal leaders like village chiefs and military officers.

These local “gatekeepers” assisted the CE team in navigating the community and complying with local codes of conduct in addition to introducing the TME programme to villagers and mobilising for villager participation. Paying respect to and consulting local leaders created an initial mutual understanding and built trust before working directly with villagers. In cases where communities were fond of their local leaders, gaining leader trust translated into gaining trust from other villagers. The head of Village C, for instance, was instrumental in generating villager participation. One senior CE team member related:

“There are two reasons behind this high participation[…]in Village C. The first reason is that the ‘community’ here would listen to only one person: the village head. He works as a village head and has been greatly supported by a strong and dedicated leader of an administrative authority of the BGF [Border Guard Force] in the area. It’s not only that he has a position as the leader, but also because he has always been through difficult times with the villagers. That’s why they have strong faith in him. Hence, the villagers would follow whatever the village head orders and would obey him”. (A CE senior team member)

The team called this stage of CE “using compasses” to navigate unfamiliar socio-political environments to more effectively engage with villagers. Simultaneously, the CE team learnt to heed villagers’ perceptions of their leaders and potential (political) tensions between leaders and the community (or some members of the community). As in the case of Village D, one CE worker elaborated:

“We need to be careful when dealing with the village head[…][here][…]because the villagers[…]are dissatisfied with him. The village head does not care about the villagers’ health at all...During my stay in this village, the village chief came and told me and other staff that the villagers’ complaints were not true...he told me that he knew very well whoever had talked bad things about him. I think that the way he talked was like a warning to the staff that they had to be careful when working in this village because he [had] known all our movements”. (A CE team member)

This dimension demonstrates how the CE team aimed not to be alienated by a single group in any locality by positioning themselves amidst contesting parties. The team needed to be aware of villagers’ affiliations (or lack thereof) with existing political power-blocs in each village. As a team member recalled what happened in Village A:

“The villagers have had to flee and struggle for their lives in the jungle for many years without getting any help from anyone. Once the situation became more peaceful, the two armed groups [KNLA and DKBA] still came to struggle for power and interests in the area. Therefore, the villagers do not feel affiliated with any group. Rather, they are disobedient. That’s why they don’t trust and don’t cooperate with outsiders. If we want to do development in this village, we can’t just go and teach people there. Instead, we need to stay with them and let them see that we are really serious [about helping them] so that we can bring them [to take part in the TME project]”. (A CE senior team member)

Moreover, the CE workers learnt that they need to keep abreast of ways in which national political processes affected their work in the villages. The “opening up of Myanmar” with the historic election in 2010 had resulted in improved relations between international organisations and the Myanmar government and greatly affected the TME programme as well as other health operations and educational projects in former war zones. Because of many decades of fighting in countless areas of Myanmar, many villagers’ deep-seated distrust towards the government had translated into distrust towards humanitarian organisations who had collaborated with the government. With the government’s attempts to implement its health and education policies throughout the country, it cooperated with international organisations such as Japan International Cooperation Agency (JICA), Save the Children, Control and Prevention of Malaria Project (CAP) and SMRU. Because the CE team was not *directly* cooperating with the government, it did not encounter the kind of challenges that those organisations cooperating with the government had met with while engaging these communities.

## Discussion

This paper summarises the approach and construction of a conceptual framework for CE where often a clear and comprehensive treatment of socio-cultural and political issues central to community participation are lacking ( Atkinson *et al*., 2011; Tindana *et al*., 2007). The nine dimensions provide a conceptual framework determined from pilot research for TME and subsequently proved integral to the subsequent scale-up of this elimination programme. The TME programme took the form of the Malaria Elimination Task Force (METF) in 2014, which has been successful in reducing malaria transmission in Karen/Kayin State of south-eastern Myanmar with nearly 60 villages participating in MDA and the establishment of over 1200 malaria posts ( König *et al*., 2018; Landier *et al*., 2017; Landier *et al*., 2018; Parker *et al*., 2017). To reach such a scale, the TME evolved into the METF, a consortium of local ethnic and community-based health care providers, which have provided services to border populations since the 1980s. Malaria posts and MDA have been central to the success of METF where CE and the nine dimensions formed the backbone of this programme as a longitudinal component underlying key METF programme components.

Whether this process of developing the nine dimensions for the TME programme can be applied to other settings remains to be seen. However, the authors argue that—although the details may differ according to context ( Atkinson *et al*., 2011)—the multidisciplinary approach outlined here is often needed for effective, scalable public health interventions in low resource settings such as along the Myanmar-Thailand border ( McNaughton, 2012). The authors, therefore, carefully point out the breadth of knowledge brought to bear by researchers involved in this programme. This team was comprised of social science researchers and community health workers with long-term knowledge and experiences regarding the socio-political realities of this heterogeneous, post-war border region.

In this vein, the authors regard this work as an important contribution to the growing literature on community engagement ( Ahmed & Palermo, 2010; Atkinson *et al*., 2011; King *et al*., 2014; Lavery *et al*., 2010; Tindana *et al*., 2007). This research fostered a peoples-centred, bottom-up approach in combination with top-down programming necessary in a region where decades of war have rendered healthcare infrastructure dilapidated and poorly functioning ( Atkinson *et al*., 2011). Construction of the conceptual framework allowed for an “investment in people” ( Atkinson *et al*., 2011) not often discussed in the health literature—that is, an investment in deep understanding of social, cultural and political realities and their effects on villagers’ quotidian lives necessary for effective communicable disease control. Often, programmes envision and plan CE activities in a trial-, project- or research-specific orientation ( Lavery *et al*., 2010), but the conceptual framework created through this research takes comprehensive stock of social and political contexts along the Myanmar-Thailand border that led to the successful scale-up of the METF programme (Cf. King *et al*., 2014).

The outcomes of this work should be placed in the context of global infectious disease control programmes and weighed against the history of successful MDA (or lack thereof). Highlighting the CE efforts in this region of long-standing social, political, ethnic and civil strife has both historical significance and precedent for malaria elimination over the past century. Malaria elimination efforts have often been hampered in areas of civil strife, as in Nicaragua, Italy and southern Europe, where widespread, early successes of malaria elimination efforts were significantly disrupted by war ( Garfield *et al*., 1989; Garfield, 1999; Majori, 2012). The TME and METF work continues to target a large, mobile population, in stark contrast to long-term efforts in the Aneityum Island of Vanuatu, which only recently achieved its goal of endemic malaria eradication after two decades of programming in a population less than 1000 persons ( Kaneko *et al*., 2014; Watanabe *et al*., 2015). Furthermore, the case of the China malaria programme, and particularly in Jiangsu Province, provide an example of malaria elimination in the context of an authoritarian regime ( Chen & Xiao, 2016; Hsiang *et al*., 2013; Hu *et al*., 2016). On-going malaria elimination efforts in China continue to struggle with border regions, mobile populations and ethnic nationalities constituting populations at higher risk of disease transmission ( Chen & Xiao, 2016; Hu *et al*., 2016). The people-centred, multidisciplinary approach applied by TME and METF in addressing significant social, political and ethnic complexities provides an example of programmatic work in a border region useful for other infectious disease programmes in similarly complex regions of the world.

This study is not without limitations. It is not powered to test CE components—individual or taken together—in reducing malaria transmission; this study instead aimed to provide a deeper narrative of social, cultural and political themes requiring consideration for successful CE. This study presents lessons learnt from qualitative, social science research, performed by health workers without significant social science training—albeit supervised by a social science researcher and guided by research assistants. In addition, interpretation of the data presented here may have been biased as views of villagers and CE team members were often translated and interpreted through senior CE team members.

## Data availability

Due to concerns that interview participants could be identified by the interview content, which could be damaging to the programme or the interview participants given the sensitive political context of the programme area if disclosed, data have not been made accessible.

## Notes

^1^There are three issues that must be settled at the outset.

Firstly, when the State Law and Order Restoration Council (SLORC) changed the country's official name to “Myanmar” in 1989, it also promoted the use or spellings of many other names in styles closer to the Burmese language, including those of ethnic nationalities. Hence “Karen” was changed to “Kayin” in most government terminology. In this article, however, the authors use “Karen/Kayin” instead of “Kayin” for three reasons: (a) “Karen” has been the preference of many Karen communities along the Myanmar-Thailand border region; (b) in keeping with villagers’ worldviews, the CE team confirmed their name as Karens, as opposed to the more official “Kayin”; (c) the authors use “Karen/Kayin” to also acknowledge the official naming of “Kayin” by successive Myanmar governments. Yet, the term “Karen” was originally used by outsiders, and its derivation is uncertain (Cf. Tangseefa, 2003).

Secondly, the term “Karen” is pluralised because this article’s treatment of the notions of identity and culture follows the philosophical and/or theoretical stances of Jacques Derrida, Gayatri Spivak, and Arjun Appadurai in the following ways. In the first place, names of all mentioned ethnicities are pluralised throughout. Such is a writing practice influenced by Derrida’s philosophy: there are always "the others within" a selfhood—no matter individual or collective—and any identity is self-differentiating (see, e.g., Derrida *et al.*, 1997). This practice is to ensure that one does not essentialise and homogenise an ethnic identity. Therefore, to avoid this conventional approach to ethnic identity, this article proposes pluralising, being aware that this naming may look "unusual”. In the second place, identity is always performative; inasmuch as identities are contingent on the performative, we use the term “Karens” as a signifier of those who enunciate and/or reenact “Karenness”—whatever this may mean during a particular historical context. In doing so, we wish to retain Spivak’s notion that identities are strategically essentialised in encounters or political struggles (see, e.g., Spivak & Grosz, 1990). In the third place, any serious understanding of culture must not ignore three key dimensions: relationality; dissensus within some framework of consensus; and weak boundaries. That is, there is no strict distinctiveness of culture because: a) who we are is always in relation to others; b) with a conceptual understanding of who we are as a collective group, there is always dissensus within our group; and c) the boundary of who we are is always weak ( Appadurai, 2013).

Thirdly, the term “peoples” is used throughout to emphasize ethnic richness that has been the norm along the Myanmar-Thailand border region. When the term is deployed as a singular, it refers to either people in general or people as a concept.

^2^At the time of the TME study, the malaria elimination activities were conducted in a postwar environment *without* armed conflict. However, armed conflict has erupted in other parts of the country following study completion. It remains to be seen, thus, whether and to what extent the conceptual framework developed in this study will be applicable to other areas where fighting has erupted intermittently.

^3^The word ‘and’ is italicised because although Myanmar’s military government was established in 1962, the histories of ethnic strife in this land—creating ethnic enmity subsequently resulting in armed conflict—had started long before the end of the Third Anglo-Burmese War in 1885 when the Kingdom of Ava fell to the British empire. Hence, when the TME started in 2013, the Karen-Burman memories of animosity had already existed for several hundreds of years (see, Tangseefa, 2003).

^4^No one can doubt Myanmar’s cultural richness. Yet, the country’s successive governments’ deployment of its ethnic composition as '135 national races' has been very problematic ( Transnational Institute and Burma Centrum Netherlands, 2014). With the country’s nation-building after over half a century of armed conflict and military government rule, no accurate statistics on any indicators is possible; data collection has been non-systematic and, for the most part, unreliable ( Oxford Burma Alliance, 2018). Such unreliability includes the country’s first national census in thirty-one years—the 2014 Population and Housing Census (see, International Crisis Group, 2014; Transnational Institute and Burma Centrum Netherlands, 2014, see also, Lidauer, 2016; Schissler, 2016). See an example of the country’s ethnic composition in Oxford Burma Alliance.

^5^Many displaced peoples had fled war and atrocities from Burma, and later, Myanmar, and resided in a string of, what the Thai state named, “temporary shelter areas” on the Thai side since their official establishment in 1984 (see, Tangseefa, 2003; Tangseefa, 2007; Tangseefa, 2016). Since Thailand is not a signatory of the 1951 Convention Relating to the Status of Refugees nor the 1967 Protocol Relating to the Status of Refugees, the term “refugee” is not part of the official lexicon of the Thai state, which designated forcibly displaced people from Burma/Myanmar as “people fleeing fighting.” Nonetheless, although the term “refugee camp” is not officially used by the Thai state’s apparatuses, it has been used by a variety of people who have come to be involved with these shelter areas.

^6^Although the CE team could speak the local language(s) and shared, in one way or another, ethno-cultural identities with the villagers, they were different from the villagers: while many of the villagers were illiterate, the CE team had a certain level of formal education with better economic and social status. Hence, the knowledge that the authors developed regarding the villagers’ understandings was, to a certain degree, “filtered” through the CE team’s perspectives.

^7^For the notion of “in-betweenness”, see Tangseefa, 2003; Tangseefa, 2006; Tangseefa, 2009.

^8^South-eastern Myanmar’s richness of natural resources attracts outsiders to villages in this part of the country. Amidst the on-going ceasefire situations as well as increasing economic activities in this region, many of which are former war zones, it is important to explore ways in which outsiders’ money and ideas have transformed target villagers to become more open.

## Supplementary material

**Supplementary File 1. Guide for semi-structured interviews conducted with the Targeted Malaria Elimination Programme Community Engagement team members.**
