# Supplementary material for: “Nine Dimensions”: A multidisciplinary approach for community engagement in a complex postwar border region as part of the targeted malaria elimination in Karen/Kayin State, Myanmar
Source: Wellcome Open Res. 2019 Feb 21;3:116. Originally published 2018 Sep 18. [Version 2] doi: 10.12688/wellcomeopenres.14698.2 (PMC6343222; doi:10.12688/wellcomeopenres.14698.2)
Supplement: Supplementary file 1 [file wellcomeopenres-3-16499-s0000.tgz › 20502cd3-0489-4816-b97c-1db74b9d9476_Suppplementary_file_-_SSI_guide.docx]

**Supplementary File 1. Guide for semi-structured interviews conducted with the Targeted Malaria Elinimation Programme Community Engagement team members.**

1. Could you tell me what have you done in this village so far as part of the TME programme?

*Probes included:* were there any problems in completing these activities? Did villagers provide any feedback about the approaches used in TME?

1. Could you tell me some characteristics of this village which you think they could become obstacles for TCE work?
2. Could you tell me your own strategy for conducting CE in this village? What was the result of that strategy?
3. Have the villagers told you whether they would like to come to take medicines?
   - Why did some decide to come, while others did not?
4. What did you do when villagers said that they did not want to come to take medicines?
5. In your point of view, does incentive play a role in convincing villagers to come and take medicines?
6. What are your thoughts about the TME situation now?
7. Could you tell me what does CE means for you?
8. What do you think about the situation of CE in this village?
9. Could you tell me your plan for CE in this village from now on?
   - Why did you plan in that way?
