# Supplementary material for: “Nine Dimensions”: A multidisciplinary approach for community engagement in a complex postwar border region as part of the targeted malaria elimination in Karen/Kayin State, Myanmar
Source: Wellcome Open Res. 2019 Feb 21;3:116. Originally published 2018 Sep 18. [Version 2] doi: 10.12688/wellcomeopenres.14698.2 (PMC6343222; doi:10.12688/wellcomeopenres.14698.2)
Supplement: Peer review history

# Peer review: Reviewer response for version 2

**DOI:** 10.21956/wellcomeopenres.16499.r34943

**Published:** 2019-02-27

**Authors:** Andrew J. Vallely

**Affiliations:** 1 New Guinea Institute of Medical Research (PNGIMR), Goroka, Papua New Guinea; 2 University of New South Wales, Sydney, Australia

I agree with the authors comments and amendments.

I have read this submission. I believe that I have an appropriate level of expertise to confirm that it is of an acceptable scientific standard.

# Peer review: Reviewer response for version 1

**DOI:** 10.21956/wellcomeopenres.16007.r34392

**Published:** 2019-01-21

**Authors:** Liwang Cui

**Affiliations:** 1 Department of Internal Medicine, University of South Florida, Tampa, FL, USA

The authors documented the significance of  community engagement (CE) in the course towards malaria elimination in a poverty-stricken, post-civil war area, along the international border. The difficulties encountered by the study team in the remote war-torn region are shared in many other places along the borders of Myanmar - extreme poverty, remoteness, political and economical marginalization, minorities, migration, mountainous terrains that are prohibitory to access, etc. A similar example is the Kachin State bordering China. For the regional malaria elimination by 2030 in the Greater Mekong Subregion, CE is essential to ensure the needed coverage. Thus, this social behavioral study provides the necessary information about the approaches taken to explore the different aspects of the nine dimensions. The authors may consider the following points:

 1. Drug resistant P. falciparum is definitely an important problem, yet P. vivax, the parasite that is difficult to eliminate, may be a more significant challenge to the regional malaria elimination. This could be mentioned in the introduction.

 2. With regard to the potential limitations in the discussion, this type of social studies could be benefited tremendously by including and training a few villagers from the study villages in the study team, which will alleviate some of the barriers that the CE team has encountered. The same may also apply to the TME work.

 3. The authors may want to discuss the prioritization of the "dimensions" based on local settings.

I have read this submission. I believe that I have an appropriate level of expertise to confirm that it is of an acceptable scientific standard.

# Response

**Published:** 2019-02-01

**Authors:** Ahmar Hashmi

Dear Dr. Cui,

Thank you for taking the time to review this work and to provide valuable suggestions. Please find our responses to your specific comments below. Please do not hesitate to leave further comments should you need clarification.

*1. Drug resistant P. falciparum is definitely an important problem, yet P. vivax, the parasite that is difficult to eliminate, may be a more significant challenge to the regional malaria elimination. This could be mentioned in the introduction.*

This is a great point. However, most of the literature to-date has focused specifically on *P. falciparum *malaria, such as the WHO expert documents outlining the current, evidence-based measures for malaria elimination cited in this study. The express purpose of the work highlighted here is targeted *P. falciparum*elimination, and we stress the specific urgency given what is currently known about drug resistance to this specific form of malaria, as well as the context within Karen/Kayin state (outlined in the “Contextual background” of the Methods section). The elimination of *P. vivax*is complicated by the need to treat the liver stages of the disease, but the knowledge acquired through CE during *P. falciparum *elimination will be used to design strategies for elimination of *P. vivax*.

*2. With regard to the potential limitations in the discussion, this type of social studies could be benefited tremendously by including and training a few villagers from the study villages in the study team, which will alleviate some of the barriers that the CE team has encountered. The same may also apply to the TME work.*

Indeed, incorporating local villagers into the CE efforts was made a central part of the Malaria Elimination Task Force (METF), which represents the expansion of the Targeted Malaria Elimination (TME) pilot work in the four villages highlighted in this study. However, when the TME study began in 2013, we were not able to train and include villagers from the four villages precisely because, as mentioned in the article, it was just a little over two years after the historic election in 2010—after being, by and large, war zones for about half a century. Furthermore, it was important to assemble a team of trained health workers: a) to explore how to fight artemisinin-resistant malaria in the four villages; and b) for the senior CE team members, specifically, to be the study’s key field researchers. Nonetheless, when METF was operationalised in 2014, subsequent to the TME pilot, this programme became successful—establishing nearly 1200 malaria posts with nearly 60 participating villages—precisely because the METF incorporated many villagers who have been applying the nine dimensions in their METF operation. 

*3. The authors may want to discuss the prioritization of the "dimensions" based on local settings.*

Thank you for this suggestion. To quote the manuscript:

"... CE workers recognized three aspects of the target villagers’ lives: worldview, time and desire. In other words, how did the villagers view their worlds and the world at large? Did villagers have time to participate in the TME programme? Did villagers desire to participate? In answering these basic questions, the following nine dimensions emerged as a crucial conceptual tool for navigating CE:..."

Therefore, we view that these three fundamental concepts—worldview, time, desire—provide a basic outline from which the nine dimensions were derived. However, the study did not aim to prioritise the nine dimension; in order to effectively and ethically conduct CE, it is imperative that health workers pay heed to all nine dimensions. The nine dimensions provide a framework that is applicable to other localities, too, where the main objective of one’s endeavour is to mobilise people in these localities to work with a CE team that may be considered “outsiders.” Worldview, time, and desire illustrate intricacies in employing CE. Contextual understanding is critical and the nine dimensions enable one to develop such an understanding. Hence, we highlight examples to further explain each of the nine dimensions, but allow for adjustments in applying this framework to different locales that may necessitate some adjustments to this framework depending upon the priorities specific to each respective context.

# Peer review: Reviewer response for version 1

**DOI:** 10.21956/wellcomeopenres.16007.r34203

**Published:** 2018-12-03

**Authors:** Andrew J. Vallely

**Affiliations:** 1 New Guinea Institute of Medical Research (PNGIMR), Goroka, Papua New Guinea; 2 University of New South Wales, Sydney, Australia

The authors describe a robust and innovative multidisciplinary approach to community engagement in a complex post-war setting.

 ​​​​It would be of interest to know whether participatory methods were considered, such as those used in participatory rural appraisal (PRA), participatory learning and action (PLA), and allied approaches. Such techniques have shown considerable value in a variety of research and development settings e.g. they are recommended in the UNAIDS Good Participatory Practice guidelines for biomedical HIV prevention trials (cf. GPP implementation toolkit: https://www.avac.org/implementation-tools). PLA tools such as listing, scoring and ranking; and/or pair-wise matrices, may have provided additional insights by enabling the CE team to explore the relationship between different dimensions from the perspective of local communities -  e.g. to understand which dimensions were considered the most important priorities in one setting, and to develop shared understanding of how and why ranked priorities might differ across local-level settings.

I have read this submission. I believe that I have an appropriate level of expertise to confirm that it is of an acceptable scientific standard.

# Response

**Published:** 2018-12-05

**Authors:** Ahmar Hashmi

Dear Professor Valley,

Thank you for your favorable review and helpful comments.

Indeed, participatory methods were considered, but the question central to this research was determining key constructs *vis-a-vis *malaria elimination within communities particular to this cross-border, post-war context that required a broader approach than more focused participatory methods would have allowed. Therefore, we elected instead on the design here to allow for a more "exploratory" approach to the issues at hand utilising the methods as we have outlined in this paper.

However, in future studies that may have a more programmatic approach, we agree with the reviewer that participatory methods and tools would prove beneficial in exploring which dimensions are more important for priority setting for future community engagement efforts.
